# Silencing of G0/G1 switch gene 2 in cutaneous squamous cell carcinoma

**DOI:** 10.1371/journal.pone.0187047

**Published:** 2017-10-26

**Authors:** Yoshimasa Nobeyama, Yoshinori Watanabe, Hidemi Nakagawa

**Affiliations:** Department of Dermatology, The Jikei University School of Medicine, Tokyo, Japan; Rudjer Boskovic Institute, CROATIA

## Abstract

**Background:**

Methylation of a CpG island (CGI; a dense cluster of CpGs) located in the 5' region of a gene suppresses that gene's transcription. The expression of G0/G1 switch gene 2 (*G0S2*) is potentially associated with tumorigenesis. The aim of this study is to elucidate the methylation status of the CGI located in the 5' region of *G0S2* (hereinafter called 5' *G0S2* CGI) in cutaneous squamous cell carcinoma (SCC).

**Methods:**

Quantitative real-time methylation-specific PCR (RT-MSP) and bisulfite sequencing were performed to evaluate the methylation statuses of cutaneous SCC and normal epithelial cell samples. Quantitative real-time reverse transcription-PCR was performed to evaluate RNA expression levels. Immunohistochemical analysis was performed to detect protein expression.

**Results:**

*G0S2* was suppressed in the five SCC cell lines with 5' *G0S2* CGI methylation levels of nearly 100.0% and was expressed in the two normal cultured keratinocytes with methylation levels of almost 0.0%. *G0S2* was re-expressed in SCC cell lines treated with a demethylating agent. The *in vivo* methylation levels of 5' *G0S2* CGI as determined by RT-MSP varied widely (0.0% to 77.7%) in 17 cutaneous SCC samples and narrowly (0.1% to 7.3%) in 6 normal epidermis samples. Nine cutaneous SCC samples exhibited higher methylation levels than the highest methylation level (7.3%) of the 6 normal epidermis samples. Bisulfite sequencing showed dense methylated CpG sites within 5' *G0S2* CGI in these highly methylated cutaneous SCC samples. The methylation levels of the cutaneous SCC samples did not correlate with any clinical parameters investigated or with histopathological grading.

**Conclusions:**

*G0S2* is silenced by aberrant DNA methylation in a subset of cutaneous SCCs.

## Introduction

DNA methylation is a DNA modification resulting from the covalent binding of a methyl group to a DNA nucleotide, such as the cytosine of a CpG dinucleotide where a 5' cytosine is adjacent to a 3' guanine [1,2]. The methylation status of individual CpG sites is faithfully copied into daughter cells [3]. CpG islands (CGIs) are dense clusters of CpGs that are often located in the 5' regions of genes. Methylation of a CGI located in the 5' region of a gene suppresses the transcription of that gene [4]. In normal cells, the CGIs located in the 5' regions of most genes are unmethylated, and these genes can be expressed [4]. However, in malignant cells, the CGIs located in the 5' regions of a number of genes, including tumor-suppressor genes, may be methylated, and the transcription of these genes is suppressed [4,5].

G0/G1 switch gene 2 (*G0S2*) is located on chromosome 1 of the human genome and encodes a small (103 amino acid) basic protein. G0S2 exerts tumor suppressive functions because G0S2 expression is required to commit cells to enter the G1 phase of the cell cycle [6,7,8,9]. Specifically, G0S2 interacts with Bcl-2 at the mitochondria, thereby disrupting formation of the Bcl-2/Bax anti-apoptotic heterodimeric complex [10]. Several studies have shown that *G0S2* is methylated *in vivo* in head and neck squamous cell carcinoma (SCC) and squamous cell lung cancer [11,12].

Cutaneous SCC is a cancer that originates from skin keratinocytes [13]. The clinical management and tumorigenesis of cutaneous SCC is clearly distinguished from other types of SCC, such as head and neck SCC, that typically arise from the mucosal epithelium of the oral cavity, oropharynx, nasal cavity and paranasal sinuses, nasopharynx, larynx, or hypopharynx [14]. Similarly, the management and properties of SCC are also distinct from squamous cell lung cancer, which typically arises from the bronchial epithelium [15,16]. The major environmental or host-dependent risk factors for cutaneous SCC are ultraviolet radiation exposure, genetic predisposition, and immunosuppression [17]. The cytogenetic changes identified in cutaneous SCC include partial chromosome gains, losses, and telomere length abnormalities [18]. Mutated genes identified in cutaneous SCC include *TP53*, *NOTCH1/2*, *CDKN2A*, *TGFBR1*, and *RAS* [18]. Tumor microenvironment abnormalities identified in cutaneous SCC include E-cadherin and type VII collagen downregulation, MMP7 overexpression, α6β4 integrin/laminin 322 over-expression, reduced expression of the co-stimulatory receptor CD40, and over-expression of the co-inhibitory receptors CTLA-4 and PD-1 [18].

In addition, aberrant DNA methylation in cutaneous SCC has been detected in several genes, including *CDKN2A* [19], *FRZB* [20], *ASC* [21], *SFRP* [22], *FOXE1* [23], *CDH13* [24] and *MiR204* [24], yet the methylation status of a putative tumor-suppressor gene, *G0S2*, in cutaneous SCC remains unexplored. The present study was conducted to assess the methylation status of the CGI located in the 5' region of *G0S2* and the expression of G0S2 in cutaneous SCC.

## Materials and methods

### Ethics statement

The ethics committee of The Jikei University School of Medicine granted approval for this study, and written informed consent for the use of tissue samples was obtained from reachable donors or their legal guardians. The ethics committee of The Jikei University School of Medicine waived the requirement for consent from unreachable donors.

### Cell lines, clinical samples, and extraction of nucleic acid

The SCC cell lines HSC-1 and HSC-5 were provided by the Japanese Collection of Research Bioresources (Tokyo, Japan). SCC cell lines A431 and DJM-1 and normal dermal fibroblasts (NB1-RGB) were provided by the Riken BioResources Center (Tsukuba, Japan). SCC cell line A388 was purchased from the American Type Culture Collection (Manassas, VA). Two normal human epidermal keratinocytes, derived from an adult (NHEKa) and a neonate (NHEKn), were obtained from ScienCell Research Laboratories (Carlsbad, CA). Seventeen paraffin-embedded SCC samples were obtained from patients (Table 1). Six normal skin samples were obtained by shaving the margins of excised epidermal cysts (Table 2). A testis tissue was obtained from testicular tumor dissected from the 29-year-old patient.

**Table 1:** Characteristics of the SCC sample donors and methylation levels of the samples.

				TNM classification				
ID	Age	Sex	Site	T	N	M	Histopathological grade	Methylation level (%)
1	61	F	nd	2	0	0	Moderately differentiated	77.4
2	62	M	Lower ext.	2	0	0	Moderately differentiated	21.9
3	86	M	Head/Neck	1	0	0	Moderately differentiated	0.1
4	56	M	Trunk	2	0	0	Well differentiated	2.1
5	80	F	Lower ext.	3	0	0	Moderately differentiated	77.7
6	96	F	Head/Neck	2	0	0	Well differentiated	15.3
7	71	M	Upper ext.	4	0	0	Moderately differentiated	3.4
8	77	F	Genitalia	2	0	0	Well differentiated	0.0
9	67	M	Genitalia	2	0	0	Well differentiated	3.1
10	83	M	Head/Neck	2	0	0	Moderately differentiated	28.3
11	76	M	Genitalia	1	0	0	Moderately differentiated	36.9
12	69	M	Genitalia	2	2	0	Moderately differentiated	1.9
13	79	M	Head/Neck	2	1	0	Poorly differentiated	8.7
14	78	F	Trunk	2	0	0	Moderately differentiated	10.7
15	83	F	Lower ext.	2	0	0	Moderately differentiated	4.0
16	90	F	Lower ext.	1	0	0	Moderately differentiated	0.0
17	79	M	Lower ext.	2	0	0	Moderately differentiated	12.8

nd, no data; age (years); F, female; M, male; ext., extremity. Age is provided in years.

**Table 2:** Characteristics of the normal epidermis sample donors and methylation levels of the samples.

ID	Age	Sex	Site	Methylation level (%)
18	68	F	Head/Neck	2.1
19	63	M	Lower ext.	2.9
20	55	M	Head/Neck	7.3
21	34	F	Trunk	0.1
22	69	M	Upper ext.	0.5
23	55	M	Trunk	2.8

nd, no data; age (years); F, female; M, male; ext., extremity. Age is provided in years.

The TNM classification of cutaneous SCC was evaluated according to the Union for International Cancer Control TNM Classification of Malignant Tumours (7^th^ edition). The diagnoses of cutaneous SCC and normal skin were made histopathologically by at least two experienced board-certified pathologists. Histopathological grading of cutaneous SCC was evaluated according to the 2006 World Health Organization Classification of Tumours, which classifies cutaneous SCCs into three grades: well differentiated SCC, moderately differentiated SCC, and poorly differentiated SCC [13]. DNA was extracted from the paraffin-embedded samples by slicing the samples into 4- to 10-μm-thick sections, deparaffinizing, then dissecting with a fine needle. Genomic DNA was extracted using a QIAamp DNA mini kit (Qiagen, Valencia, CA). Total RNA was isolated using ISOGEN (Nippon Gene, Tokyo, Japan).

### Treatment with 5-aza-2'-deoxycytidine

For 5-aza-2'-deoxycytidine (5-aza-dC; Sigma-Aldrich, St Louis, MO) treatment, SCC cells were seeded at a density of 1.0 × 10^5^ to 2.5 × 10^5^ cells per 10-cm dish at day 0, then exposed to medium containing 1.0 μM 5-aza-dC at days 1 and 3 for a total exposure time of 96 h. Cells were harvested at day 5. For each cell line, 5-aza-dC-treated cultures showed mild growth suppression on day 5 when compared to the corresponding untreated cells.

### Quantitative real-time methylation-specific PCR (RT-MSP) and bisulfite sequencing

An EZ DNA Methylation-Gold kit (Zymo Research, Irvine, CA) was used according to the manufacturer's instructions to treat 1.0 μg of *Bam*HI-digested genomic DNA from each cell line or clinical sample with sodium bisulfite, then the purified bisulfite-treated DNA was dissolved in 40 μl of TE buffer.

**Fig 1:**
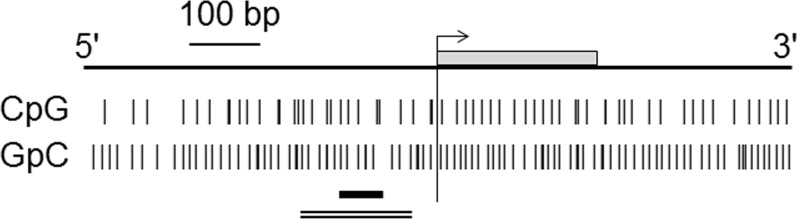
Structure of the 5' region of the *G0S2* gene. The gray square indicates exon 1. The long vertical line indicates the transcriptional start site. The short vertical lines indicate individual CpG sites (upper) and GpC sites (lower). The analyzed regions are shown as a single horizontal line for RT-MSP and as a double horizontal line for bisulfite sequencing.

For RT-MSP, 1.0 μl of bisulfite-treated DNA solution was used as the template and amplified using a 7500 Real-Time PCR System (Applied Biosystems, Foster City, CA) and SYBR Green PCR Master Mix I (Toyobo, Osaka, Japan) mixed with each of two reaction mixtures: one that contained a primer set specific to the methylated DNA sequence, and one that contained a primer set specific for the unmethylated DNA sequence. The sequences of the methylated DNA-specific primer set were 5'-CGTTGCGATGGTATTCGCGTC-3' (forward primer) and 5'-ACGCGCTAAACACGCTCCG-3' (reverse primer), and were designed to amplify from 140 bp to 80 bp upstream of the major *G0S2* start site (based on the *G0S2* sequence in NC_000001.11) (Fig 1). The sequences of the unmethylated DNA-specific primer set were 5'-GGTTATGTGTTGAGTATGTTTT-3' (forward primer) and 5'-CCACACACCCCACTACA-3' (reverse primer), and were designed to amplify from 150 to 76 bp upstream of the major *G0S2* start site. The number of molecules of a specific gene in a sample was quantified by comparing the amount of amplification product with that of standard samples containing 10^1^ to 10^8^ template copies of the gene. The methylation level was defined as the number of methylated molecules divided by the total number of methylated and unmethylated DNA molecules. DNA methylated with the *Sss*I methylase (New England Biolabs, Beverly, MA) was used as the methylated DNA control under specific amplification conditions with the methylated DNA-specific primer set. DNA amplified with the GenomiPhi DNA amplification kit (GE Healthcare Bioscience, Little Chalfont, UK) was used as the unmethylated DNA control under specific amplification conditions with the unmethylated DNA-specific primer set.

For bisulfite sequencing, 1.0 μl of sodium bisulfite-treated DNA was used for PCR with primers common to the methylated and unmethylated DNA sequences. The sequences of the primer set were 5'-GAAAAGGAGGGGGTGGAAT-3' (forward primer) and 5'-AACTCCRAATCCTCCCCTAC-3' (reverse primer), in which R indicates A or G. The primer set was designed to amplify from 210 to 34 bp upstream of the major *G0S2* start site. The PCR products were cloned into a cloning vector and 12 clones were cycle-sequenced for each sample.

### Quantitative real-time reverse transcription-PCR (RT-PCR)

Total RNA was treated with DNase I (Ambion, Austin, TX), then a Superscript II kit (Life Technologies, Rockville, MD) was used to synthesize cDNA from 1.0 μg of total RNA. RT-PCR was performed using SYBR Green PCR Master Mix I (Toyobo) and a 7500 Real-Time PCR System (Applied Biosystems). The sequences of the primer set were 5'-ACTTCAGAGAAACCGCTGAC-3' (forward primer) and 5'-TGTCATGACAATGCAGTGCT-3' (reverse primer). The number of molecules of a specific gene in a sample was quantified by comparing the amount of the amplification product with that of standard samples containing 10^1^ to 10^8^ template copies of the gene. The quantity of RNA of each gene was normalized to that of the glyceraldehyde-3-phosphate dehydrogenase gene (*GAPDH*).

### Immunohistochemical analysis

Immunohistochemical staining of G0S2 protein was performed using a rabbit polyclonal antibody against human G0S2 (Atlas Antibodies, Voltavägen, Sweden) as the primary antibody. Deparaffinized sections were incubated with 100-fold-diluted primary antibody at 4°C for 24 hours, then with the secondary antibody (anti-rabbit IgG in Histofine Simple Stain MAX-PO system (R); Nichirei, Tokyo, Japan) at room temperature for 40 min. Binding of the secondary antibody was visualized using Histofine Simple Stain diaminobenzidine solution (Nichirei). Slides were counterstained with hematoxylin. Omission of the primary antibody provided the negative control, and absence of staining was confirmed. Staining of testicular tissue was used as the positive control, and the presence of staining was confirmed.

### Statistical analysis

Statistical analysis was performed using the commercially available software SPSS version 18 (SPSS Japan, Tokyo, Japan). Significant differences in laboratory data were assessed via linear regression analysis, the Mann-Whitney U test, or the Kruskal-Wallis test. *P* < 0.05 was considered to be statistically significant.

## Results

### The methylation status of the 5' region of *G0S2* CGI regulates the transcription of *G0S2*

**Fig 2:**
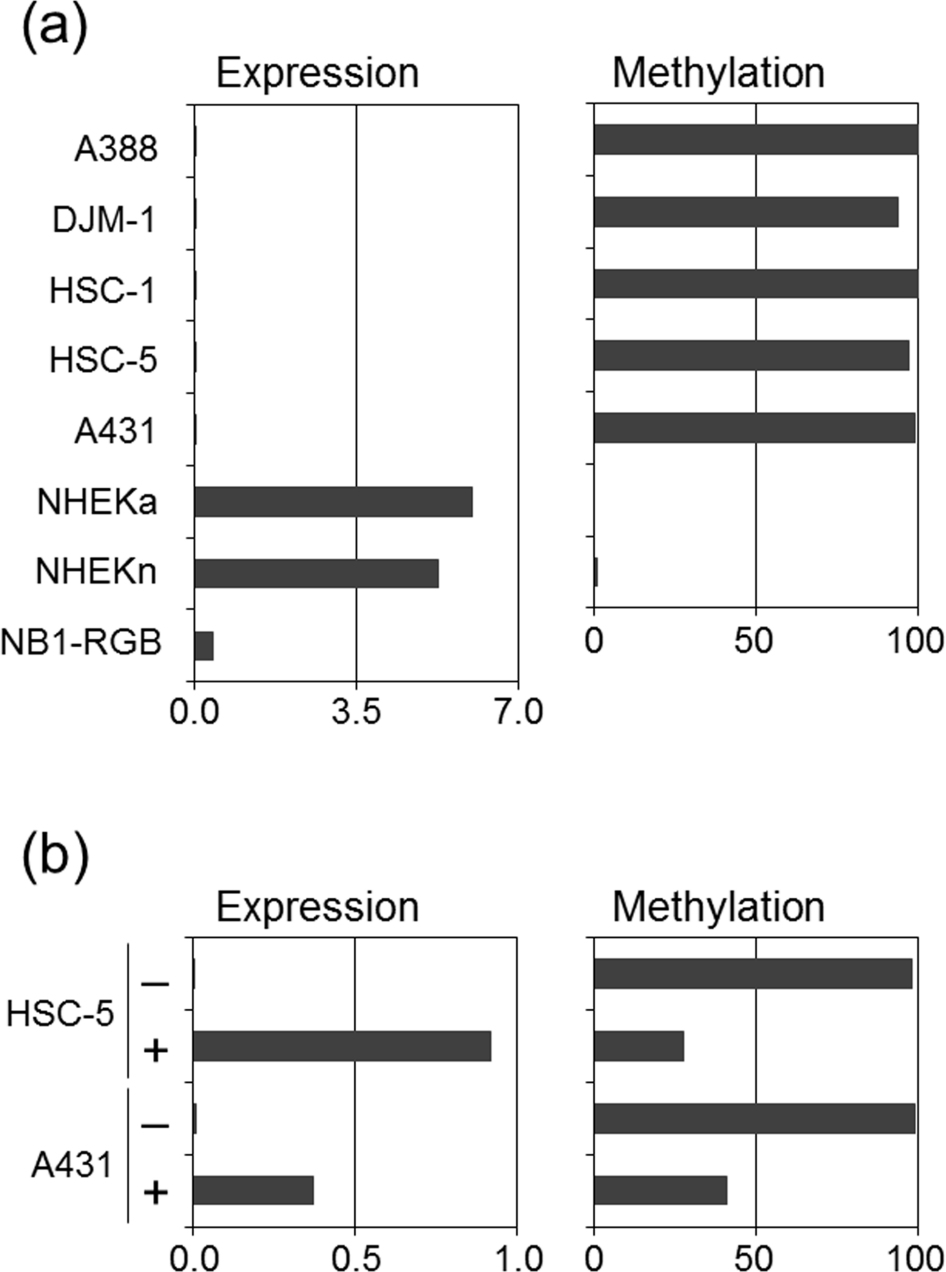
Regulation of *G0S2* expression by the methylation status of the CGI located in the 5' region of *G0S2*. **(a)** The expression levels of *G0S2* and the methylation levels of the CGI located in the 5' region of *G0S2*. The bar graph on the left indicates the expression levels of *G0S2* divided by that of *GAPDH* (×10^−2^) in the SCC cell lines, normal cultured keratinocytes (NHEKa and NHEKn), and normal cultured dermal fibroblasts (NB1-RGB). The bar graph on the right indicates the methylation level (%) of the 5' *G0S2* CGI in the SCC cell lines and normal cultured keratinocytes (NHEKa and NHEKn). Both bar graphs correspond to each other with respect to the cell lines and normal cultured keratinocytes. **(b)** Induction of *G0S2* expression in HSC-5 and A431 cells after treatment with a demethylating agent. The bar graph on the left indicates the expression levels of *G0S2* divided by that of *GAPDH* (× 10^−2^) in HSC-5 and A431 cells with or without treatment with the demethylating agent. The bar graph on the right indicates the methylation level (%) of 5' *G0S2* CGI in HSC-5 and A431 cells with or without treatment with the demethylating agent. Both bar graphs correspond to each other with respect to the cells. Plus and minus signs indicate cell lines treated with the demethylating agent and those that were untreated, respectively.

Potential associations between the methylation status of the 5' region of *G0S2* CGI (hereinafter termed 5' *G0S2* CGI) (Fig 1) and the transcription of *G0S2* were analyzed by performing RT-PCR and RT-MSP for five SCC cell lines (A388, DJM-1, HSC-1, HSC-5 and A431) and normal cultured keratinocytes (NHEKn and NHEKa). RT-PCR showed gene suppression in all five SCC cell lines, and RT-MSP detected few or no DNA molecules unmethylated at the 5' *G0S2* CGI in all cell lines (the methylation level was 100.0% in A388, 93.7% in DJM-1, 100.0% in HSC-1, 96.9% in HSC-5, and 98.8% in A431 cells). In contrast, RT-PCR showed *G0S2* expression in normal keratinocytes, and RT-MSP detected a substantial amount of *G0S2* CGI unmethylated at the 5' region (the methylation level was 0.0% in NHEKa and 0.8% in NHEKn) (Fig 2A). Treatment of the highly methylated and *G0S2* suppressed cells A388 and HSC-5 with the demethylating agent 5-aza-dC resulted in transcriptional activation of *G0S2* and decreased methylation levels in the 5' *G0S2* CGI (Fig 2B).

### High methylation levels are indicated in cutaneous SCCs but not in normal skins

**Fig 3:**
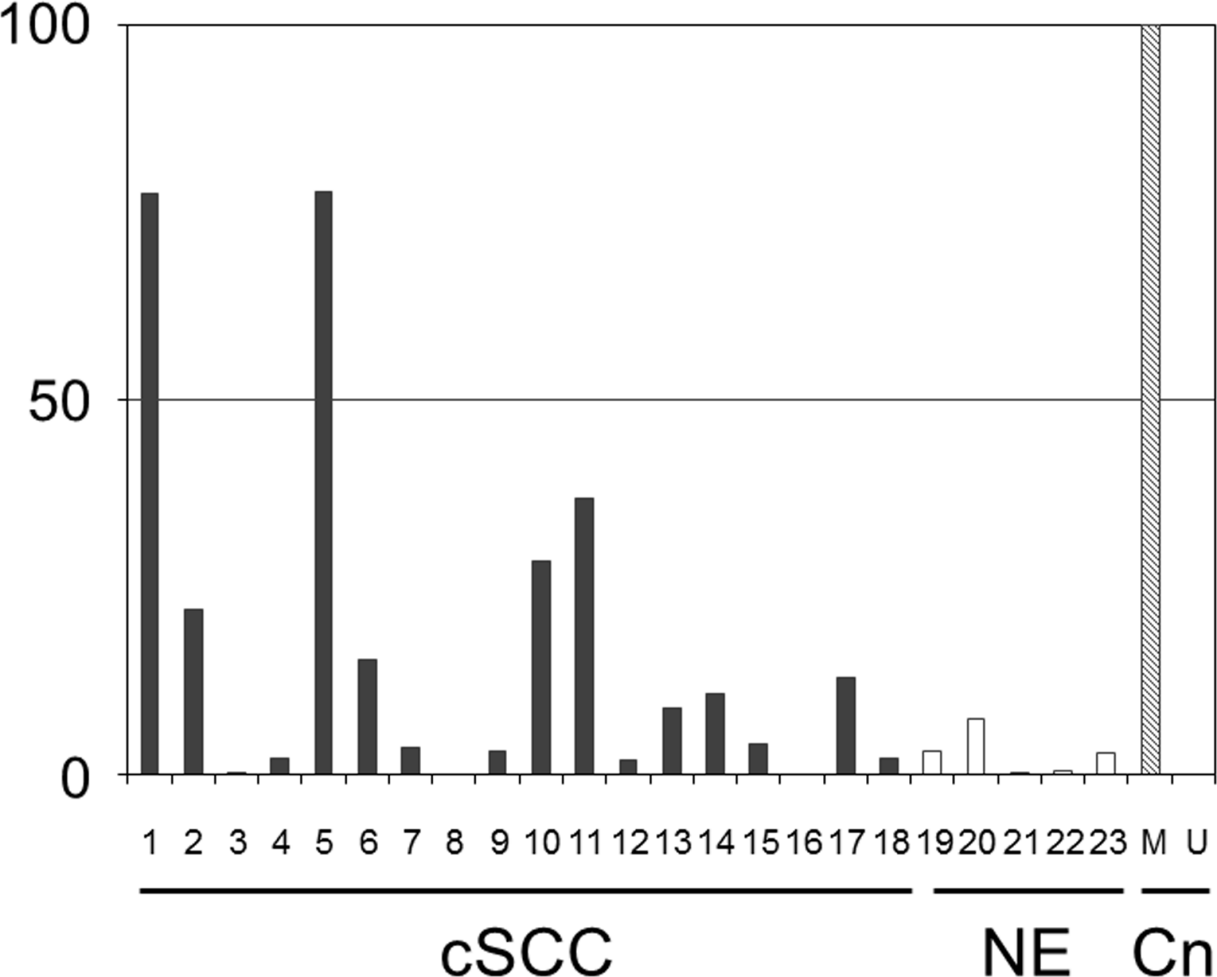
The methylation levels of the CGI located in the 5' region of *G0S2* in 17 clinical cutaneous SCC samples (cSCC, #1 to #17) and six normal epidermis samples (NE, #19 to #23). The vertical and horizontal rows of numbers indicate the methylation level (%) and each sample ID, respectively. M and U indicate the experimental controls (Cn) of methylated control DNA and unmethylated control DNA, respectively.

The methylation levels of 5' *G0S2* CGI were assessed *in vivo* by performing RT-MSP for 17 clinical cutaneous SCC samples and six normal epidermis samples. RT-MSP showed a wide range of methylation levels of 5' *G0S2* CGI in the cutaneous SCC samples, from 0.0 to 77.7% (Table 1 and Fig 3). Representative samples include 77.7% methylation in sample #5, 77.4% in #1, 36.9% in #11, 28.3% in #10, and 21.9% in #2. In contrast, the assay showed a narrow range of methylation levels of 5' *G0S2* CGI in the normal epidermis samples (ranging from 0.1% to 7.3%; Table 2 and Fig 3).

### The bisulfite sequencing data are generally consistent with the RT-MSP data

**Fig 4:**
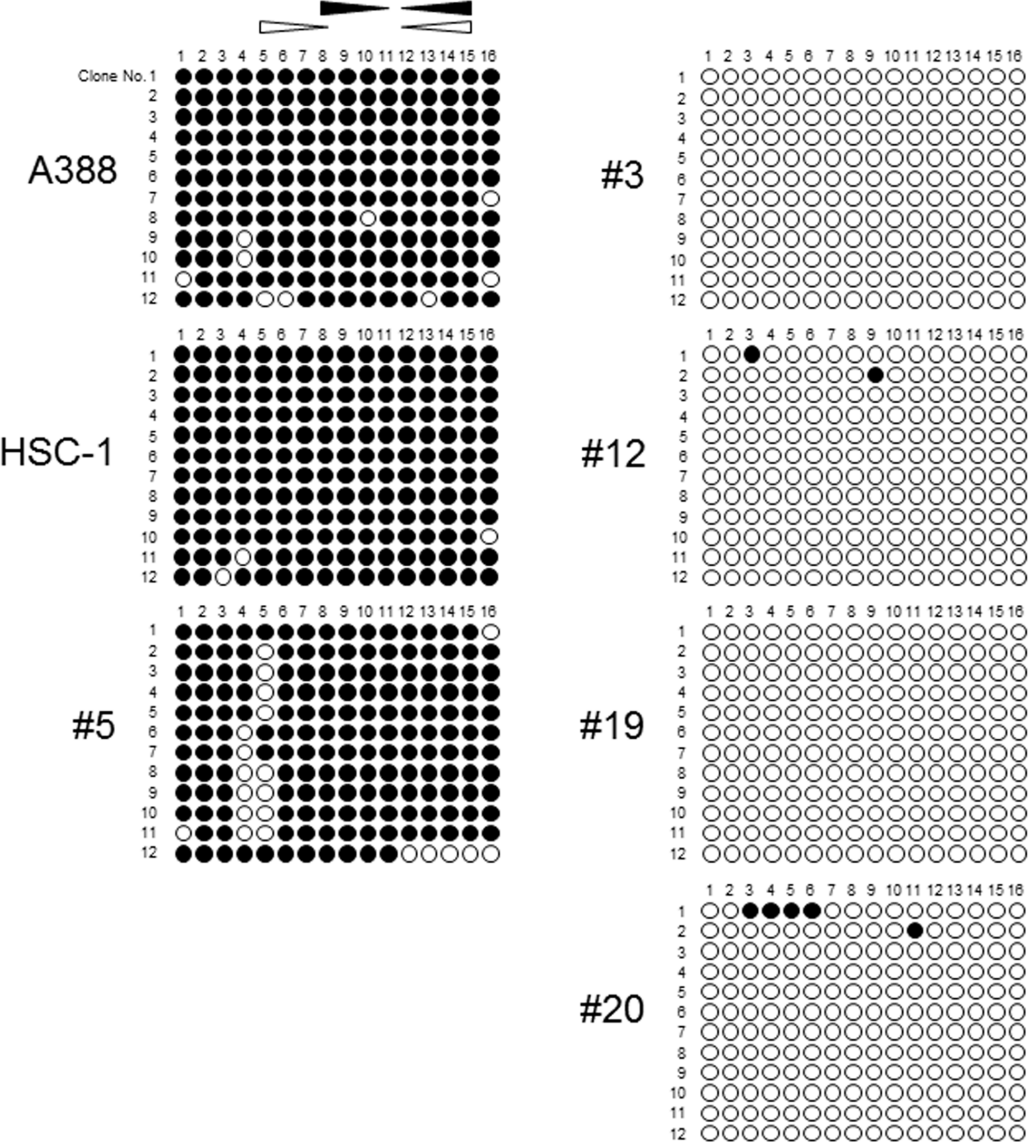
The results of bisulfite sequencing of the CGI located in the 5' region of *G0S2* in representative clinical samples. Black and white circles indicate the methylated and unmethylated CpG sites, respectively. Black and white triangles indicate the location of the RT-MSP primer set specific to the methylated and unmethylated DNA sequences, respectively. The horizontal row of numbers indicates the positions of the CpG sites. The vertical column of numbers indicates each clone.

The methylation status determined by RT-MSP was confirmed by bisulfite sequencing for seven representative samples from the two groupings based on the RT-MSP findings: SCC samples A388, HSC-1, and #5 (high methylation levels), and SCC samples #3 and #12 and normal epidermis samples #19 and #20 (low methylation levels). The bisulfite sequencing data were generally consistent with the RT-MSP data: the CpG sites within 5' *G0S2* CGI were densely methylated in samples A388, HSC-1 and #5, but essentially unmethylated in samples #3, #12, #19 and #20 (Fig 4).

### G0S2 is suppressed in cutaneous SCC samples with methylated 5' *G0S2* CGI

The suppression of G0S2 protein in cutaneous SCC samples with high methylation levels of 5' *G0S2* CGI was assessed by immunohistochemical analysis of the cutaneous SCC samples with high methylation levels and the results showed suppression of G0S2 protein in these samples (Table 1 and S1 Fig).

### Methylation levels do not correlate with any clinical parameters investigated

Statistical analyses were performed to assess potential associations between methylation status and individual clinical parameters (S1 Table). The methylation levels of 5' *G0S2* CGI did not correlate with any clinical parameters investigated, including age, sex, sampling site, T-classification, N-classification, and histopathological grading in patients with cutaneous SCC.

## Discussion

The present study demonstrated for the first time that *G0S2* is silenced by aberrant methylation of the CGI located in the 5' region of *G0S2* (5' *G0S2* CGI) in cutaneous SCC. Previous studies showed that *G0S2* is methylated in head and neck SCC and in squamous cell lung cancer [11,12]. Consequently, 5' *G0S2* CGI can apparently be methylated in any histopathological subtype of SCC derived from different tissues.

The data from *in vitro* examinations demonstrated (i) a clear difference in methylation levels: namely, high methylation levels in all SCC cell lines and very low methylation levels in all normal keratinocytes tested, (ii) SCC cell lines with few or no unmethylated 5' *G0S2* CGI molecules exhibited suppression of that gene, and (iii) SCC cell lines with few or no unmethylated DNA molecules exhibited gene re-expression after treatment with a demethylating agent. These observations indicate that *G0S2* can be silenced by aberrant methylation in 5' G0S2 CGI, at least in SCC cell lines. These *in vitro* data prompted us to analyze the methylation status of *in vivo* cutaneous SCCs and normal keratinocytes.

The *in vivo* examination of normal epidermis samples revealed methylation levels ranging from 0.1% to 7.3%. If the cut-off value is set at the highest methylation value of 7.3% in these normal samples, then 9/17 cutaneous SCC samples exhibit abnormally high methylation levels. On the other hand, the *in vitro* examination data showed that all five of the SCC cell lines exhibited high methylation levels of 5' *G0S2* CGI. There is therefore a discrepancy between the *in vivo* and *in vitro* data for the frequency of high methylation levels in cutaneous SCC samples. This discrepancy may be due to the *in vitro* culture conditions of SCC cells promoting the methylation of 5' *G0S2* CGI.

The bisulfite sequencing data were compatible with the RT-MSP data. Bisulfite sequencing detected the detailed methylation statuses of 5' *G0S2* CGI: representative clinical samples with high methylation levels as determined by RT-MSP exhibited dense methylated CpG sites in the CGI, while those with low methylation levels as determined by RT-MSP exhibited sparse methylated CpG sites (Fig 4). These data indicate that RT-MSP is a reliable procedure for evaluating the methylation status of 5' *G0S2* CGI.

A limitation of this study is that only 17 cutaneous SCC samples were analyzed and thus evidence from the statistical analysis data of this small number of samples is relatively weak. Another limitation is that G0S2 protein expression analyses for cutaneous SCC samples and normal skin samples with low levels of methylation were not performed because appropriate samples were not available. This should be addressed in future studies.

In conclusion, the methylation status of the CGI located in the 5' region of *G0S2* regulates the expression of *G0S2* in cutaneous SCC. *G0S2* is silenced by aberrant DNA methylation of 5' *G0S2* CGI in a subset of cutaneous SCCs.

## Supporting information

S1 Table Calculated *P*-value for each clinical parameter for the SCC samples. (DOCX)

S1 Fig Immunohistochemical analysis of G0S2 protein suppression in representative cutaneous SCC samples with high 5' G0S2 CGI methylation levels. Cutaneous SCC samples #1 and #5 were representatives of samples with high methylation levels of 5' *G0S2* CGI. NC indicates a negative control. Omission of the primary antibody was used as the negative control, and absence of staining was confirmed. PC indicates a positive control. The anti-G0S2 antibodies reacted with androcytes in normal testis tissue. (All samples, ×400).
(TIF)
