# Supplementary material for: Silencing of G0/G1 switch gene 2 in cutaneous squamous cell carcinoma
Source: PLoS One. 2017 Oct 26;12(10):e0187047. doi: 10.1371/journal.pone.0187047 (PMC5658152; doi:10.1371/journal.pone.0187047)
Supplement: S1 Table — (DOCX) [file pone.0187047.s001.docx]

**S1 Table.** Calculated *P*-value for each clinical parameter for the SCC samples

| Factors | *P*-value | Statistical analysis |
| --- | --- | --- |
| Age | 0.551 | Linear regression analysis |
| Sex | 0.813 | Mann-Whitney U test |
| Site | 0.635 | Kruskal-Wallis test |
| T classification | 0.332 | Kruskal-Wallis test |
| N classification | 0.592 | Mann-Whitney U test |
| Hisopathological grade | 0.421 | Kruskal-Wallis test |
