# Supplementary figures and images for: Silencing of G0/G1 switch gene 2 in cutaneous squamous cell carcinoma
Source: PLoS One. 2017 Oct 26;12(10):e0187047. doi: 10.1371/journal.pone.0187047 (PMC5658152; doi:10.1371/journal.pone.0187047)

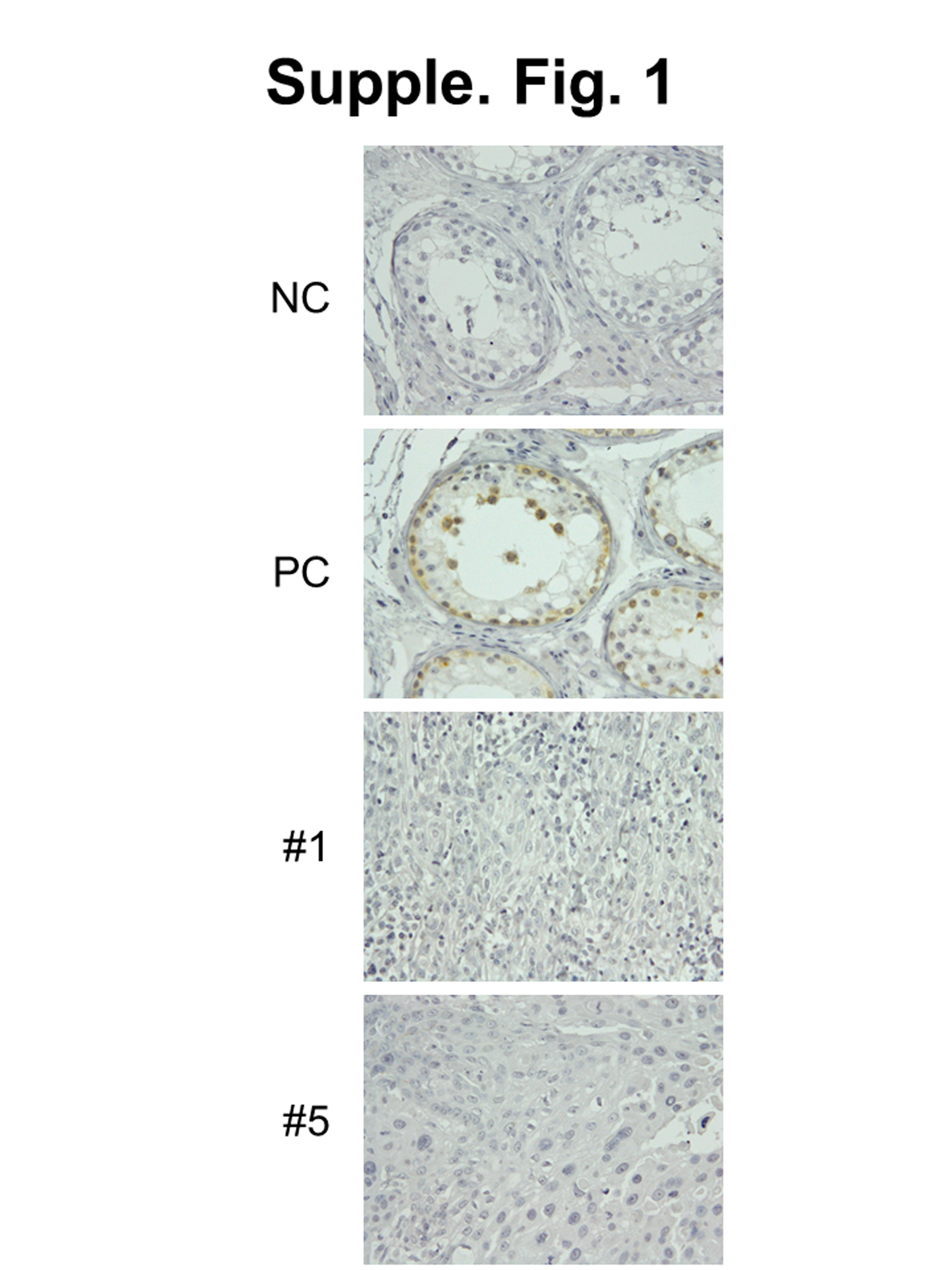

Supplement: S1 Fig — Cutaneous SCC samples #1 and #5 were representatives of samples with high methylation levels of 5' G0S2 CGI. NC indicates a negative control. Omission of the primary antibody was used as the negative control, and absence of staining was confirmed. PC indicates a positive control. The anti-G0S2 antibodies reacted with androcytes in normal testis tissue. (All samples, ×400). (TIF) [file pone.0187047.s002.tif]
